# Exceptional Response of Metastatic Chromophobe Renal Cell Carcinoma to Vascular Endothelial Growth Factor (VEGF) Inhibitors: Should Increased VEGF-C Expression Be Used to Guide Treatment?

**DOI:** 10.1155/2019/2479823

**Published:** 2019-12-28

**Authors:** Jacob W. Bruinius, Karl J. Dykema, Sabrina L. Noyes, Bin Tean Teh, Brian R. Lane

**Affiliations:** ^1^Spectrum Health, Grand Rapids, MI, USA; ^2^Helix Scribes Solutions, Grand Rapids, MI, USA; ^3^Van Andel Research Institute, Grand Rapids, MI, USA; ^4^National Cancer Centre Singapore, Singapore; ^5^Michigan State University College of Human Medicine, Grand Rapids, MI, USA

## Abstract

There is sparse literature demonstrating effective treatments for metastatic chromophobe renal cell carcinoma (ChRCC). The tyrosine kinase inhibitor (TKI) sunitinib selectively inhibits the VEGF pathway and it is a standard care for metastatic clear cell renal cell carcinoma (ccRCC), although data supporting its use in ChRCC is much more limited. A 56-year-old underwent palliative nephrectomy for locally-advanced ChRCC with sarcomatoid differentiation. Tumor gene expression profiling using Affymetrix HG-U133 Plus 2.0 GeneChip platform demonstrated significantly elevated VEGF-C expression compared to normal renal tissue (*n* = 12) and other types RCC (*n* = 158). Adjuvant sunitinib was used to treat his residual unresectable retroperitoneal lymph nodes. He demonstrated an exceptional response and underwent complete surgical resection four months later. He has been managed with TKIs for nearly nine years with only minimal disease progression. Additional studies exploring treatment options for patients with non-clear cell RCC are needed; in their absence, we would recommend TKIs for patients whose tumors bear a similar molecular profile.

## 1. Introduction

With 73,820 new cases estimated to be diagnosed in 2019, kidney cancer is one of the most frequently diagnosed forms of cancer in the United States [1]. Most of these (≥90%) were diagnosed as renal cell carcinoma (RCC), which includes a diverse classification of cancers originating from the epithelial cells of the renal tubule [2]. The three most common forms of RCC are clear cell (ccRCC), papillary, and chromophobe (ChRCC) which respectively account for 65%, 20%, and 5% of all reported cases of RCC [3]. ChRCC generally carries a more favorable prognosis compared to other histologic subtypes of RCC, with a five-year survival rate >90% [4]. Paradoxically, median progression-free survival (PFS) is reduced with metastatic ChRCC compared to metastatic clear cell RCC (ccRCC) [5]. Currently, there is limited data available to guide the medical management of metastatic nccRCC, as registration trials of targeted therapies have either excluded nccRCC in favor of the more common diagnosis of ccRCC, or enrolled nccRCC in numbers too small to make definitive recommendations [6]. There are no guideline-recommended systemic therapies for ChRCC at present, leaving treatment to physician discretion.

Elucidation of the molecular pathogenesis of RCC has led to the development of targeted therapies for patients who initially present with advanced disease or who experience recurrence after primary treatment [7]. Sunitinib is a small-molecule tyrosine kinase inhibitor (TKI) of vascular endothelial growth factor (VEGF), which drives tumor angiogenesis [8]. Other strategy approaches involve the inhibition of mammalian target of rapamycin (mTOR) or immunotherapy based on the programmed cell death protein 1 (PD-1). Each of these targeted therapeutic approaches offers increased efficacy when compared to the previous standard of care (high-dose interleukin-2) and boast a more tolerable toxicity profile [2].

Over the past 12 years several additional TKIs have also demonstrated activity in RCC including sorafenib, pazopanib, axitinib, and cabozantinib [7]. These have proven efficacious in prolonging PFS in patients with ccRCC, although a recent study was published which found that nccRCC patients treated with TKIs have worse clinical outcomes compared to matched ccRCC patients, with a median PFS of 11.8 months versus 6.5 months (*p* = 0.018) in ccRCC and nccRCC patients, respectively [9]. In this report, we describe the molecular profile of a patient who presented with advanced ChRCC and demonstrated an exceptional response to sunitinib with continued TKI therapy for nearly nine years.

## 2. Case Presentation

**Figure 1:**
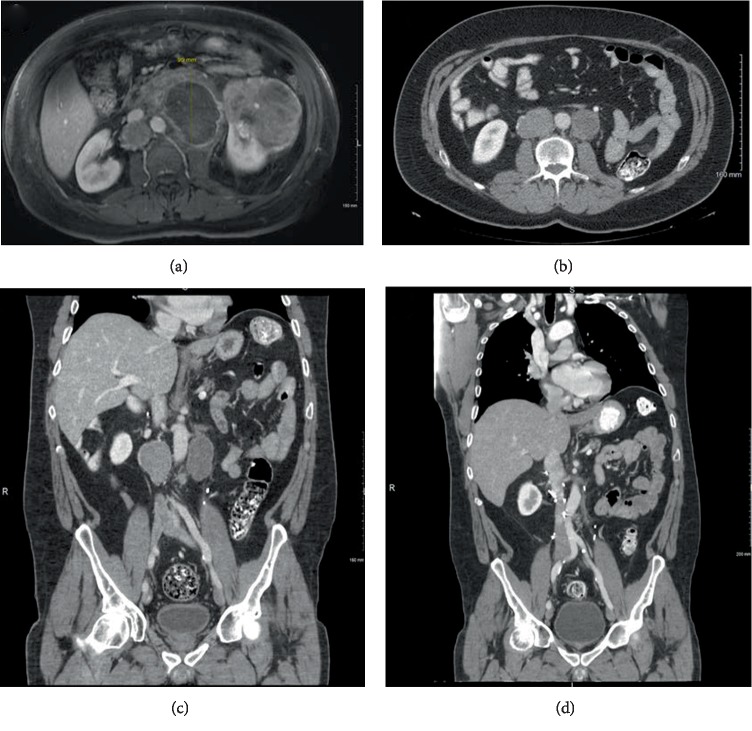
Magnetic resonance imaging (MRI) showing the large renal mass and metastatic lymph nodes. Dramatic reduction in tumor volume in an enlarged lymph node as demonstrated by MRI taken at initial presentation (a) vs. after 5 months of sunitinib (b) and (c). After retroperitoneal lymph node dissection, a complete response was obtained (d).

A 56-year-old man presented with complaints of generalized body aches, back pain, and bilateral flank pain. Noncontrast computed tomography (CT) imaging revealed a 13.3 × 12.3 × 10.4 cm left renal mass and multiple enlarged retroperitoneal lymph nodes (LN), including 11.9 × 10.1 × 8.7 cm necrotic para-aortic LN and 5.3 × 4.4 × 4.0 cm interaortocaval LNs. Subsequent CT of the head, neck, and thorax were negative for metastases. Magnetic resonance imaging (MRI) revealed significant necrotic components in the tumor with no venous tumor thrombus (Figure 1(a)). Pathology from renal mass biopsy indicated an eosinophilic renal neoplasm in favor of non-clear cell RCC (nccRCC).

The patient underwent open left radical nephrectomy within two weeks of initial presentation. During surgery, the large necrotic LNs were densely adherent to the duodenum and small bowel mesentery and determined to be unresectable. Surgical pathology described a 16.0 × 13.0 × 12.5 cm pT3a ChRCC with prominent sarcomatoid features (grade 4) and positive medial surgical margins. Immunohistochemistry revealed one component with plant-like tumor cells that were positive for CK7 and C-KIT and negative for CD10 and a spindle sarcomatoid tumor component positive for CD10 and vimentin.

Based on the histopathologic results, the patient was treated with a 4/2 schedule of sunitinib 50 mg once daily. After four cycles, the para-aortic LN had shrunk by 91% (Figures 1(b) and 1(c)). Complete retroperitoneal LN dissection 7 months after nephrectomy included, a grossly necrotic para-aortic LN (6.0 × 4.0 × 3.8 cm) and golden, nonnecrotic interaortocaval LN (6.0 × 5.0 × 4.2 cm).

After LN dissection (Figure 1(d)) the patient achieved a continued response on sunitinib and continued therapy for another 23 months (28 months total). Side effects of systemic therapy were minimal, apart from fatigue noted after 16 months of treatment. An enlarged left mediastinal LN (1.5 cm) was noted 21 months after presentation and eventually grew to 2.1 cm over the next 8 months. After resection of this pathologically-confirmed metastatic ChRCC, sunitinib was discontinued and he started second-line therapy with axitinib (5 mg daily) which continued for 36 months without disease progression. He discontinued therapy for 2 months due to fatigue, but resumed TKI therapy with pazopanib (800 mg daily) when imaging revealed progression of a retro-aortic LN to 1.7 cm. He continued on pazopanib for 22 months with only minimal disease progression, before switching to cabozantinib (40 mg Mon−Fri), which was continued for 17 months. During this time, he received intensity modulated radiation therapy (4500 cGy) to the retro-aortic LN which had grown to 2.1 cm. Notably, the patient has achieved an ongoing prolonged response on systemic anti-VEGF therapy for nearly nine years.

## 3. Results

**Figure 2:**
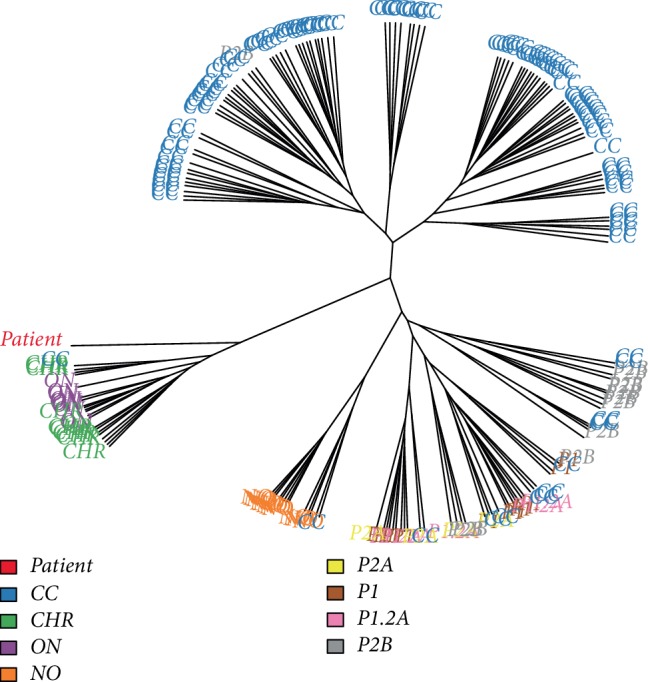
Unrooted cluster tree comparing the index tumor (*Patient*) to normal renal tissue (*NO*, *n* = 12), chromophobe RCC (*CHR*, *n* = 10), oncocytoma (*ON*, *n* = 14), clear cell RCC (*CC*, *n* = 102), papillary type 1 RCC (*P1*, *n* = 5), papillary mixed type 1/type 2a RCC (*P1.2A*, *n* = 4), papillary type 2a RCC (*P2A*, *n* = 4), and papillary type 2b RCC (*P2B*, *n* = 1) based on the 500 most variable genes.

The patient's tumor underwent gene expression profiling using the Affymetrix HG-U133 Plus 2.0 GeneChip platform. This data was compared to normal renal tissue (*n* = 12) as well as multiple forms of RCC (*n* = 158) publicly available within the Gene Expression Omnibus database [10]. The data were read using Bioconductor [11] and processed using the Affymetrix [12] and MBNI custom CDF packages [13]. The whole gene expression matrix is attached as a Supplementary [Supplementary-material supplementary-material-1]. To determine the molecular relationship between the patient and the other renal samples, a unrooted cluster tree was plotted based on 500 most variable genes from the dataset (Figure 2). The patient's tumor clustered with the other oncocytic neoplasms (ChRCC and oncocytoma), but was the most divergent of these tumors.

**Figure 3:**
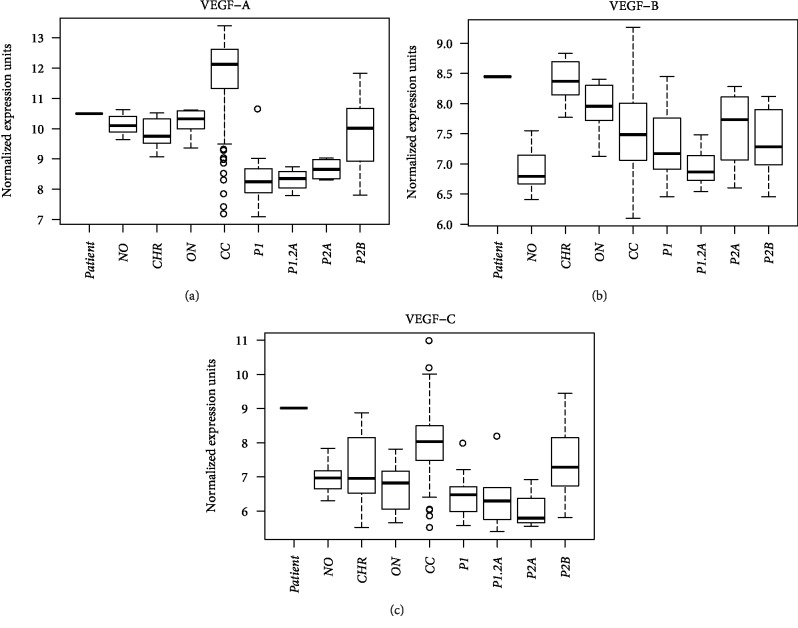
VEGF expression levels of the exceptional responder. Normalized gene expression levels of VEGF-A (a), VEGF-B (b), and VEGF-C (c) measured in the patient's tumor (*Patient*) compared to normal renal tissue (*NO*), chromophobe RCC (*CHR*), oncocytoma (*ON*), clear cell RCC (*CC*), papillary type 1 RCC (*P1*), papillary mixed type 1/type 2a RCC (*P1.2A*), papillary type 2a RCC (*P2A*), and papillary type 2b RCC (*P2B*).

Gene expression of VEGF-A, VEGF-B, and VEGF-C were determined for a range of renal epithelial neoplasms, including 102 ccRCC, 14 papillary RCC, 10 ChRCC, 14 oncocytoma, and 12 normal kidney controls. Normalized gene expression levels of VEGF-A were significantly elevated compared to all other subgroups (Figure 3). In contrast, VEGF-B and VEGF-C expression varied greatly in ccRCC, with a range of values overlapping that of the other renal epithelial neoplasms. The ChRCC and oncocytoma samples had similar expression of VEGF-A, VEGF-B, and VEGF-C, with greater variability of VEGF-C expression among the ChRCC samples.

Patient VEGF-C expression was significantly elevated (9.0) when compared to the 10 ChRCC tumor samples (median 6.9) and normal renal tissue (median 6.9). The 10 ChRCC samples had a wide range of VEGF-C expression (5.6–8.8) as did the 102 ccRCC samples (5.7–11.1), indicating wide heterogeneity of expression in these tumors. The patient's tumor also displayed increased VEGF-B expression (8.4) compared with normal renal tissue (median 6.7) although the range of expression among the 10 ChRCC overlapped the test patient (median 8.3, range 7.7–8.7). In contrast, VEGF-A expression (10.6) was similar to the levels seen in other ChRCC (median 9.7), oncocytoma (median 10.5), and normal renal tissue (median 10.1).

## 4. Discussion

To date there are limited clinical trial data [6] and few long-term responses documented for patients with metastatic ChRCC [14], none of which have included any molecular analysis of the responders. While ChRCC commonly involves extensive chromosomal losses (Y, 1, 2, 6, 10, 13, 17, and 21), it is generally regarded as a particularly indolent subtype of RCC [15]. This is intriguing, as excessive aneuploidy can compromise cellular proliferation, increasing the potential for cancer metastasis [16]. Comprehensive molecular profiling of 66 ChRCC tumors listed in The Cancer Genome Atlas (TCGA) database observed recurrent genomic structural arrangements involving the *TERT* promoter region and elevated *TERT* expression, as well as, diffusely increased mitochondrial function and mitochondrial DNA alterations [17]. Another comprehensive genomic analysis of nccRCC by Durinck et al. examined 49 ChRCC tumors and found *TP53*, *PTEN*, *FAAH2*, *PDHB*, *PDXDC1*, and *ZNF765* to be significantly mutated relative to normal tissue; this is in contrast to ccRCC, which is characterized by mutations in *VHL*, *TCEB1*, *PTEN*, *PBRM1*, *SETD2*, *BAP1*, *KDM5C*, *MTOR*, *PIK3CA,* and *TP53* relative to normal tissue [18]. The fundamental molecular differences between ccRCC and nccRCC, and ChRCC in particular, have profound clinical importance, particularly when these cancers require systemic therapy. Despite the lack of evidence and differences in tumor histology, most patients with metastatic nccRCC are treated with the same targeted therapies that are used for ccRCC. Choueiri et al. examined a cohort of 53 patients with nccRCC, including 12 with ChRCC. Of those, 3 (25%) achieved a response on TKI therapy and the median PFS for the entire group was 10.6 months. Phase II clinical trial data from RECORD-3 [19], ESPN [20], and ASPEN [21] have shown sunitinib to be slightly more efficacious than everolimus in treating metastatic nccRCC, although this effect is modest at best. Conclusions that can be drawn from these studies are limited as they do not indicate results according to the varying histology of each nccRCC subtype. A systematic review and meta-analysis examining systemic treatment options for patients with nccRCC concluded that VEGF-targeted therapies were slightly favored over mTOR inhibitors, although this did not reach the level of statistical significance [22]. A more recent cross-channel group study examining anti-angiogenic therapy vs. mTOR inhibitors for metastatic ChRCC came to a similar conclusion [23]. Immunotherapy has also recently emerged as another option to treat metastatic RCC, and clinical trials that compare TKI therapy vs. immunotherapy are currently underway, although these studies have only enrolled patients with ccRCC [24].The role of immunotherapy in treating nccRCC, for now, remains an open question.

Certain genomic features have been identified which may assist in predicting clinical outcomes for patients with metastatic ChRCC. Casuscelli et al. examined genomic features specific to 35 metastatic ChRCC tumors and found that mutations to *TP53* and *PTEN, *and imbalanced chromosome duplication in ≥3 chromosomes, were associated with inferior clinical outcomes [25]. A recent report examining differentially expressed genes within the TCGA database found that two such genes, *SKA1* and *ERCC6L*, were associated with improved overall survival in patients with ChRCC [26]. While these data could certainly be used to guide further research into the genomic background of ChRCC, their immediate clinical utility is limited as they do not point towards any specific treatment option. The outcome of patients with ChRCC with sarcomatoid differentiation have poorer prognosis, as is the case for other RCC subtypes with sarcomatoid features [27]. Indeed, since variability of clinical outcomes are largely determined by tumor heterogeneity, the diverse genomic landscape of ChRCC (and nccRCC in general) better lends itself towards precision medicine, which can deliver tailored targeted therapies exploiting the specific subtype of cancer expressed in a given patient [28]. This is an emerging field and no predictive biomarkers are currently available to guide patients towards particular therapies [2].

Our data suggest VEGF-C could be utilized as a clinically viable biomarker to guide patients with ChRCC towards TKI therapy. While a major limitation of this study is that our data comes from a single patient, examinations of exceptional responders provide a unique opportunity to generate hypotheses which may prove useful in elucidating key molecular mechanisms behind disease processes. These so-called “N of 1” case reports have been previously used to uncover new cancer treatment options [29] and the nation's leading cancer research centers have been systematically collecting data on exceptional responders to guide future work on targeted therapies and drug discovery [30].

## 5. Conclusion

In this report, we report a patient diagnosed with ChRCC bearing increased expression of VEGF-C and VEGF-B who exhibited a prolonged response to sunitinib and has been stable on anti-VEGF therapies for nearly nine years. We feel that TKI's could be effective for other patients with ChRCC, particularly for patients whose tumors bear a similar molecular profile. Additional studies exploring potential treatment options for patients with nccRCC may help identify efficacious treatments for these uncommon cancers.

## Supplementary Materials

Supplementary Materials Supplementary Table 1: Gene expression log2 fold change versus normal renal tissue.
